# The Function of the Mitochondrial Calcium Uniporter in Neurodegenerative Disorders

**DOI:** 10.3390/ijms18020248

**Published:** 2017-02-10

**Authors:** Yajin Liao, Yuan Dong, Jinbo Cheng

**Affiliations:** 1 The Brain Science Center, Beijing Institute of Basic Medical Sciences, No. 27 Taiping Road, Haidian District, Beijing 100039, China; lyajin@163.com; 2 The State Key Laboratory of Brain and Cognitive Sciences, Institute of Biophysics, Chinese Academy of Sciences, Beijing 100101, China; 3 Department of Biochemistry, Qingdao University Medical College, Qingdao 266071, China; juliadong829@hotmail.com

**Keywords:** MCU, neurodegenerative disorder, inflammation, iron overload, oxidative stress

## Abstract

The mitochondrial calcium uniporter (MCU)—a calcium uniporter on the inner membrane of mitochondria—controls the mitochondrial calcium uptake in normal and abnormal situations. Mitochondrial calcium is essential for the production of adenosine triphosphate (ATP); however, excessive calcium will induce mitochondrial dysfunction. Calcium homeostasis disruption and mitochondrial dysfunction is observed in many neurodegenerative disorders. However, the role and regulatory mechanism of the MCU in the development of these diseases are obscure. In this review, we summarize the role of the MCU in controlling oxidative stress-elevated mitochondrial calcium and its function in neurodegenerative disorders. Inhibition of the MCU signaling pathway might be a new target for the treatment of neurodegenerative disorders.

## 1. Introduction

Calcium, which can be referred to as the “life and death signal”, plays an important role in multiple biological process [1]. Changes in calcium concentration in the cytoplasm or mitochondria are proven to be involved in many fundamental biological processes such as neuronal activation, adenosine triphosphate (ATP) production and cell death. Calcium homeostasis is critical for the function of the central nerve system. Disruption of calcium homeostasis in the brain is found in multiple neurodegenerative disorders, such as Alzheimer’s disease (AD), Parkinson’s disease (PD), Huntington disease (HD), amyotrophic lateral sclerosis (ALS) and cerebral ischemic stroke [2,3,4,5]. Mitochondria are able to take up calcium rapidly and, therefore, play an essential role in regulating cellular calcium homeostasis [6,7,8]. The mitochondrial calcium level has been proven to be involved in a series of physiological and pathological process. Calcium flows into mitochondria and can activate three critical enzymes of the Krebs cycle (pyruvate, α-ketoglutarate, and isocitrate dehydrogenases) and increase mitochondrial reactive oxygen species (ROS) levels, which facilitates ATP production [9,10,11,12,13]. Calcium overload in mitochondria leads to an excess in the production of ROS, causing mitochondrial dysfunction, which triggers complement-dependent superoxide-mediated programmed cell death and other biological pathways [14].

To describe how mitochondria accomplish calcium influx and efflux, several channels and uniporters have been identified. Among those, the mitochondrial calcium uniporter (MCU) is one of the most important and highly selective calcium transporting complexes in mitochondrial calcium uptake and is located on the inner membrane of mitochondria [15]. Despite intensive studies on the MCU, its encoding genes were only recently identified. According to a recent study, the MCU is comprised of several parts, where Mcu functions as the pore-forming and calcium-conducting subunit, while MICU1 (calcium uptake protein 1, mitochondrial), MICU2 (calcium uptake protein 2, mitochondrial), EMRE (essential MCU regulator, mitochondrial), MCUR1 (mitochondrial calcium uniporter regulator 1) and MCUb (calcium uniporter regulatory subunit MCUb, mitochondrial) work as regulatory subunits [16]. Here, in our review, we summarize the function and regulatory mechanisms of the MCU in the brain and emphasize the perplexing questions on the MCU that need to be answered in future studies.

## 2. The Molecular Components and Calcium Transporting Mechanism of the MCU

**Figure 1:**
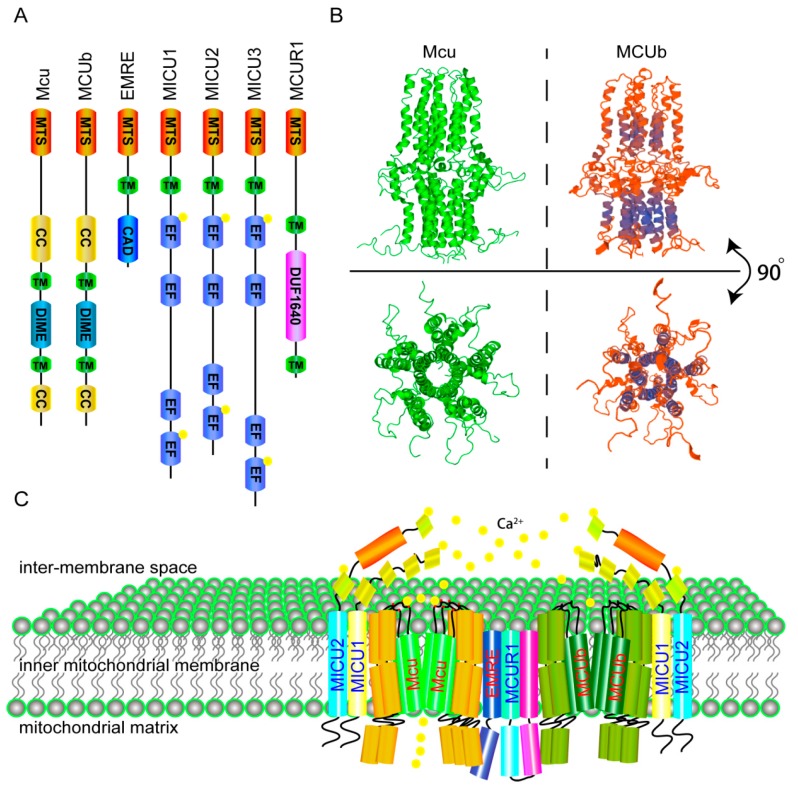
The molecular components and structure of mitochondrial calcium uniporter (MCU). (**A**) The components of *Homo sapiens* MCU. From left to right, the schematic shows a linear overview of the predicted domain architecture of each of the seven *Homo sapiens* MCU components. MTS: mitochondrial targeting signal, TM: transmembrane domain, CC: coiled-coil domain, DIME: the conserved DIME motif, CAD: carboxy-terminal domain, EF: EF-hand domain, DUF1640: protein of unknown function (DUF1640); (**B**) The structure of Mcu-∆NTD (left) and MCUb-∆NTD (right). Upper: front view of Mcu and MCUb show both of them have three distinct layers, down: top view displays the calcium pore formed by five transmembrane domains of Mcu (left) or MCUb (right) (http://swissmodel.expasy.org/repository/) [25,28]; (**C**) Cartoon of *Homo sapiens* MCU in the inner membrane of mitochondria.

The calcium uniporter is located on the inner membrane of mitochondria. Its function can be inhibited by some small molecules, such as ruthenium red and RU360. Recently, the composition of the mitochondrial calcium uniporter was identified [16,17,18,19]. MICU1 is encoded by *Micu1*, which is the first identified subunit. As an EF-hand domain-containing protein, MICU1 is located on the inner membrane of mitochondria and plays a regulatory role in mitochondrial calcium uptake [20]. Following the discovery of MICU1, the crucial pore-forming protein Mcu (encoded by *MCU*) was discovered using an integrative genomics approach [19]. Since then, the remaining subunits of the MCU have been identified: MICU2 (*EFHA1*), MICU3 (*EFHA2*), MCUb (*CCDC109B*), MCUR1 (*CCDC90A*) and EMRE (*Smdt1*) (Figure 1A) [21,22,23,24]. Those subunits interact with Mcu and MICU1 and form a complex that regulates the calcium homeostasis of mitochondria.

Among the MCU subunits, Mcu is the critical pore-forming subunit. Structure analysis reveals that Mcu is a homo-oligomer with two transmembrane domains. The second transmembrane helixes from five Mcu form a hydrophilic pore in the inner membrane of mitochondria, and the DXXE motif forms the pore entrance in the inter-membrane space (Figure 1B, left and Figure 1C) [25]. Mutations of key acidic residues in the motif (D261A or E264A) abolish the calcium uptake activity of the MCU [19]. In addition, the S259A mutant form of Mcu completely blocks the effect of Ru360 [19]. However, the Mcu deficiency phenotype in mice is dependent on mouse strains. Mice from mixed genetic backgrounds are normal, while mice of inbred strains cannot live without Mcu [26,27]. MCUb is the negative regulatory subunit of the MCU. A recent study discovered that MCUb contains two transmembrane domains and forms a structure similar to the Mcu [22]. Homology modeling of MCUb by Swiss model shows MCUb has the same three-dimension-structure and hydrophilic pore with Mcu (Figure 1B, right) (http://swissmodel.expasy.org/repository/) [28]. However, substitutions of the amino acids in the pore forming region result in lost activity of calcium intake [22]. Overexpression of MCUb results in a decreased calcium uptake activity of the MCU, indicating that MCUb is a dominant-negative pore-forming subunit. MICU1 is an important gatekeeper of the MCU and is found to localize to the mitochondrial matrix side of the inner membrane of mitochondria. MICU1 controls MCU-mediated mitochondrial calcium influx by interacting with the coiled-coil domains of Mcu on its N-terminal [29]. Crystal structure analysis indicates that Ca^2+^-free MICU1 forms a hexamer. When binding with calcium, MICU1 reforms to oligomers and activates MCU [30]. Loss of MICU1 in cells leads to an adaptive mitochondrial matrix calcium accumulation and increased resting mitochondrial calcium level [31,32]. Deletion of MICU1 in mice causes significant perinatal mortality, marked ataxia and muscle weakness. In addition, patients with loss-of-function mutations in MICU1 exhibit an obvious phenotype with brain and muscle disorders. Individuals with MICU1 mutations display proximal myopathy, learning difficulties and a progressive extrapyramidal movement disorder [33]. MICU2 is another gatekeeper of the MCU and plays nonredundant roles with MICU1 in inhibiting the calcium intake activity of the MCU, when outside calcium is low and permitting MCU transport of calcium into mitochondria in response to a stimulus above the threshold [31]. MICU3 plays the same role as MICU2 but has a different expression pattern compare with MICU2. EMRE is a 10-kD single-pass transmembrane protein that functions as a positive regulator of MCU [24]. EMRE is first synthesized as a precursor, which is processed into mature form by general mitochondrial processing peptidase MPP (MPP) with the help of m-AAA protease interacting protein 1 (MAIP1). Mature EMRE inserts into mitochondrial inner membrane, while excess precursor EMRE is degraded [34]. According to studies in yeast, the tight interaction between EMRE and the MCU is essential for mitochondrial calcium uptake. Additionally, EMRE is required for the MCU to reconstitute the minimal uniporter activity in yeast [35]. Assembly assay further shows that loss of EMRE impairs the assembly of Mcu subunits, and overexpression of EMRE facilitates the formation of MCU complex. In addition, both precursor and mature EMRE are capable of forming active calcium uptake channel with Mcu, but only mature EMRE ensures the assembly of gatekeeper subunits to prevent calcium overload. The gatekeeper decreased form MCU is observed in m-AAA proteinase deficient neurons, which has increased calcium influx into mitochondria and more sensitivity to cell death [34]. Those results indicate that EMRE may function as a structural factor for the opening of the Mcu-forming pore and a recruiter for the gatekeeper. MCUR1 was first found to be required for MCU-mediated mitochondrial calcium uptake. One recent study shows that MCUR1 functions as a scaffold factor in the MCU complex by binding to Mcu and EMRE [36]. Cells with MCUR1 knockdown/overexpression display the same mitochondrial calcium uptake activities as the MCU knockdown/overexpression cells [23]. However, one study demonstrated that MCUR1 is a cytochrome *c* oxidase assembly factor and does not directly regulate the MCU [37]. Thus, the role of MCUR1 in the MCU needs further studies. Collectively, all those subunits form the MCU complex in the inner membrane of mitochondria to maintain mitochondrial calcium homeostasis (Figure 1C) [38].

## 3. The Role of the MCU in Excitotoxicity

**Figure 2:**
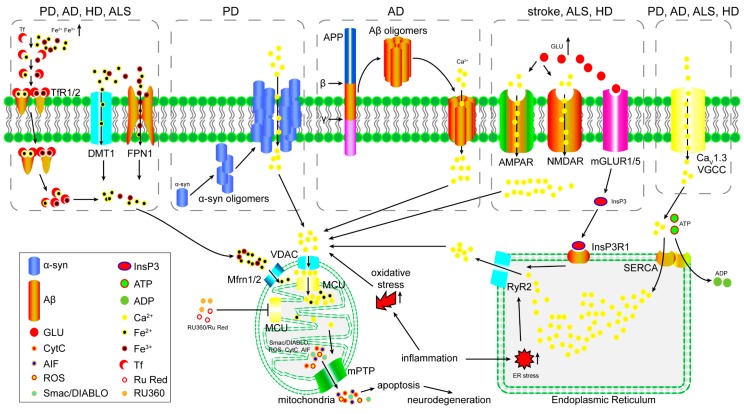
The proposed model of mitochondrial calcium dysregulation in neurodegenerative disorders and the potentiality of MCU as therapeutic target. Iron over-imported by TfR1/2, DMT1 and FPN1 will be taken up by mitochondria through Mfrn1/2 and potentially MCU. Excess calcium gets into cytoplasm through Aβ oligomers or α-syn oligomers formed ion channel, or glutamate receptors in pathological tissues of neurodegenerative disorders. InsP3R1 activation induce calcium release from ER. Continuous activation of L-type calcium channel Ca_V_1.3 VGCC in PD, AD, HD and ALS. Excess calcium and iron in cytosol induce mitochondrial dysfunction, mPTP opening and pro-apoptosis factors release through MCU-mediated calcium and iron uptake. (PD: Parkinson’s disease; AD: Alzheimer’s disease; HD: Huntington’s disease; ALS: Amyotrophic lateral sclerosis; TfR1/2: transferrin receptor protein 1/2; DMT1: divalent cation transporter 1; FPN1: ferroportin-1; AMPAR: α-amino-3-hydroxy-5-methyl-4-isoxazolepropionic acid receptor; NMDAR: N-methyl-D-aspartate receptor; mGLUR1/5: metabotropic glutamate receptor 1/5; VGCC: voltage-dependent L-type calcium channel subunit β-2; VDAC: Voltage-dependent anion-selective channel; Mfrn1/2: mitoferrin 1/2: MCU: mitochondrial calcium uniporter; RyR2: ryanodine receptor 2; InsP3R1: inositol 1,4,5-trisphosphate receptor type 1; SERCA: Sarcoplasmic/endoplasmic reticulum calcium ATPase 2; GLU: glutamate; mPTP: mitochondrial permeability transition pore; CytC: cytochrome C; ROS: reactive oxygen species; AIF: apoptosis-inducing factor; Smac/DIABLO: Second mitochondria-derived activator of caspase/direct inhibitor of apoptosis-binding protein with low pI. ER: endoplasmic reticulum).

Excitotoxicity is the pathological process in which neuronal cells are damaged or killed by overloaded stimulation by neurotransmitters [39]. Among the diverse transmitters in the mammalian central nervous system, glutamate is the major excitatory neurotransmitter and plays a critical role in the development of AD, PD and stroke [40,41,42,43]. To date, every glutamate receptor subtype discovered has been proven to mediate neurotoxicity [44,45,46]. An obvious phenomenon in glutamate-induced excitotoxicity is delayed calcium deregulation (DCD). A secondary, irreversible increase in calcium occurs after a variable latent period post glutamate stimulation, which precedes and predicts subsequent cell death [47]. According to recent studies, most glutamate receptor-mediated neurotoxicity has been proven to be calcium-dependent, and selective inhibition of glutamate receptors could block the prolonged elevation of calcium [48,49]. In addition, glutamate receptors have found to be capable of calcium uptake (Figure 2) [50,51]. Besides direct calcium transportation, some glutamate receptors were found to regulate inositol 1,4,5-trisphosphate (InsP3) receptor and L-type Ca^2+^ channel, and subsequently cause calcium increasing in cytosol (Figure 2) [52,53]. Except glutamate-induced excitotoxicity, calcium is also implicated in estrogenic chemical-induced neurotoxicity [54]. Excessive elevation in the intracellular calcium level and mitochondrial dysfunction have been observed in neurons in an excitotoxic condition, although the role of mitochondrial calcium in excitotoxicity has not been clearly described [55,56]. In addition, glutamate-induced mitochondrial dysfunction and loss of membrane potential could be suppressed by RU360, suggests that mitochondrial calcium uptake is essential for excitotoxicity (Figure 2) [57]. A recent study revealed that the MCU controls excitotoxicity and is implicated in N-methyl-D-aspartate (NMDA) receptor-mediated cell death [58]. In this study, authors found that the activation of the NMDA receptor leads to an increase in the mitochondrial calcium level and promotes mitochondrial depolarization. Overexpression of Mcu leads to an NMDA-mediated increase in the mitochondrial calcium level and causes a loss of the mitochondrial membrane potential. In contrast, knockdown of Mcu decreases NMDA-mediated mitochondrial calcium levels, therefore preventing the loss of the mitochondrial membrane potential and excitotoxicity cell death. Those results indicate that the MCU may play an essential role in excitotoxicity; however, more studies are needed to confirm the function of the MCU in vivo.

## 4. The Role of the MCU in Iron Overload-Induced Mitochondrial Dysfunction

Iron overload is an inducer of brain mitochondrial dysfunction, and excess accumulation of iron in the brain has been detected in various neurodegenerative disorders, such as PD, AD and HD (Figure 2) [59,60,61,62,63,64]. Iron is taken up by cells in transferrin-bound form or free form. In neuron, iron is imported in transferrin-iron (Tf-iron) form and is mediated by transferrin receptor protein 1 (TfR1) (Tf-Fe^2+^) or TfR2 (Tf-Fe^3+^). When Tf-iron-TfR complex was endocytosed, iron would be released in cytosol. In TfR1/2-deficient cells, iron is mainly taken up by metal transporters divalent metal transporter 1 (DMT1) and ferroportin-1 (FPN1) in free form (Figure 2) [65]. Overexpression of those transporters is sufficient to induce iron overload and cell death, in addition, increased expression of DMT1 had been observed in PD and AD murine model [66,67]. Accumulation of iron in cytoplasm will induce excessive iron influx into mitochondria through mitoferrin 1/2 (Mfrn1/2) and cause mitochondrial dysfunction [68]. So, individuals with iron overload usually display miscellaneous signs and symptoms. Brains from rats fed with excess iron show severe meningeal hemorrhage, congestion, edema and lipid peroxidation, whereas, 5-HT and DOPA are dramatically decreased [69]. These results indicate that iron overload is sufficient to induce neurodegenerative disease. Excess iron causes cell injury mainly by uncoupling oxidative-phosphonates and inducing mitochondrial dysfunction, resulting in increased reactive oxygen species (ROS) production and oxidative-stress [70,71]. Evidence from in vitro experiments suggests that the MCU may be involved in iron overload-induced mitochondrial dysfunction. Recent studies have shown that only the MCU blocker RU360 could completely prevent iron overload-induced cardiac or brain mitochondrial dysfunction by decreasing ROS production, mitochondrial depolarization and swelling. However, the mitochondrial permeability transition pore (mPTP) blocker cyclosporin A (CsA) merely inhibits ROS production [72,73,74]. These results indicate that the MCU could be a major portal for mitochondrial iron uptake; however, the mechanism demands further study because there is no direct evidence that the MCU could transport iron into mitochondria.

## 5. The Role of the MCU in Oxidative Stress-Induced Mitochondrial Dysfunction

Oxidative stress is an accelerator of neurodegenerative diseases and is a main factor in PD and stroke [75,76,77]. Cytosolic calcium overload is a common consequence of oxidative stress and indicates that excess cytosolic calcium may induce mitochondrial calcium overload and dysfunction [78,79]. An increase in the calcium level is reported to be an important indicator for oxidative stress-associated diseases. Patients with high serum calcium have been found to be more likely to suffer from a poor short-term prognosis and higher risk of long-term mortality after ischemic stroke [80]. Results from in vitro experiments indicated that inhibition of the activity of the MCU by the MCU inhibitor RU360 or siRNA or miRNA against Mcu rendered the cells resistant to oxidative stress. Consistently, overexpression of the MCU can cause higher sensitivity to the oxidative stress of cells [81,82]. In addition, inhibition of the MCU reduces oxygen glucose deprivation/reperfusion (OGD/RP)-induced autophagy and mitophagy, therefore improving cell viability after OGD/RP [83]. Results from in vivo studies also prove that in a middle cerebral artery occlusion and reperfusion (MCAO-R) model, compared with normal or spermine-treated rats, rats treated with RU360 show decreased total infarct volume, reduced water content, less neuronal damage and cell apoptosis, and lower aquaporin 4 expression [84]. However, another study demonstrated that oxidative stress does not increase mitochondrial calcium in the first few minutes, while cytosolic calcium rapidly rises [85]. These conflicts may be due to the fact that the increase in the mitochondrial calcium lags far behind the increase in the cytosolic calcium, which is similar to DCD (Figure 2).

## 6. The Function of the MCU in Inflammation

Inflammation is a critical factor in the development of neurodegenerative disorders, such as PD, AD, ALS, depression, and stroke [86,87,88]. Recent studies have shown that upon activation of the inflammatory response, the production of ROS increases, causing damage to the blood-brain barrier (BBB) and mitophagy activation, together with elevated expression of inflammatory cytokines [89,90,91,92]. Mitochondrial calcium homeostasis is reported to be disrupted in infectious diseases, indicating that mitochondrial calcium may be involved in inflammation-associated diseases (Figure 2) [93,94,95,96]. To date, evidence has indicated that the MCU plays an important role in the regulation of bacteria- and virus-induced activation of inflammation. In the *Pseudomonas aeruginosa*-dependent inflammatory response, the MCU-mediated increase in mitochondrial calcium is critical for the activation of NACHT, LRR and PYD domain-containing protein 3 (NLRP3) and interleukin 1 β (IL-1β) and interleukin 18 (IL-18) processing [97]. The MCU can integrate pro-inflammatory signals and relay the information to NLRP3 by increasing mitochondrial calcium uptake and inducing mitochondrial dysfunction. Another study showed that MCU-mediated calcium uptake is essential for the virus-induced increase in endoplasmic reticulum (ER) stress, which facilitates the amplification of retinoic acid-inducible gene I (RIG-I)-mediated activation of the type I interferon pathway and apoptosis [98]. Taken together, the MCU functions as an enhancer in inflammation by increasing the calcium concentration of mitochondria and upregulation of ER stress. However, the mechanism by which the MCU increases mitochondrial calcium uptake in inflammation is not clear.

## 7. The Potential of the MCU as a Target for Neurodegenerative Disorder Therapy

Calcium overload can be induced by a wide range of stimuli [99,100]. Mitochondrial dysfunction and calcium homeostasis disruption are observed in many other neurodegenerative disorders as well [2,3,4,5,101,102,103,104].

Alzheimer’s disease is a neurodegenerative disease with specific accumulation of Alzheimer’s β-amyloid (Aβ_1–42_) peptides in the brain tissue of AD patients [105]. Mitochondrial dysfunction is one of the major symptoms in AD. Studies show that Aβ_1–42_ peptides accumulate in the mitochondria of the brain in AD patients and APP-transgenic mice [106,107]. Aβ_1–42_ peptides are imported into the mitochondria via the translocase of the outer membrane import machinery and localizes to mitochondrial cristae [107]. The translocation of Aβ_1–42_ peptides into mitochondria has been linked to mitotoxic effects by in vitro and in vivo evidence, such as increased release of adenylate kinase and ROS, and this mitotoxicity is promoted by aging [108,109,110]. Calcium influx is one of the mitotoxic effects induced by Aβ_1–42_, and evidence suggests that oxidative stress and the subsequent neurodegeneration induced by accumulation of Aβ_1–42_ peptides are calcium influx-dependent (Figure 2) [111,112,113]. This is suitable to support the Aβ ion channel hypothesis. The Aβ ion channel hypothesis suggests that Aβ oligomers form a non-specific ion channel on cell membrane and mitochondrial membrane [114]. This hypothesis had been supported by some other evidences. Three-dimensional structure analysis revealed that Aβ oligomers could form a channel in bilayer, and another study showed the channel display a strong selective affinity for Calcium (Figure 2) [115,116]. Therefore, the “modulation of mitochondrial calcium as a pharmacological target for Alzheimer’s disease” has been recently proposed [117].

PD is another major neurodegenerative disease with extensive mitochondrial dysfunction and oxidative stress [118]. The pathological hallmark of PD is Lewy bodies, of which the primary structural component is abnormal aggregated α-synuclein (SNCA) [119]. In vivo experiments indicate that oligomeric SNCA is sufficient to induce mitochondrial calcium overload and mitochondrial dysfunction (Figure 2) [120,121]. Similar to Aβ oligomers, SNCA oligomers could also form an ion channel on lipid membrane and lead to calcium dyshomeostasis (Figure 2) [122]. Mitochondrial dysfunction and oxidative stress is also observed in genetic forms of PD. Mutations in PINK1, DJ1 and LRRK2 are the main genetic factors of familial PD [123,124,125,126]. All three genes have been demonstrated to have an effect on mitochondrial function. Deficiencies in these genes make mitochondria more vulnerable to oxidative stress [127,128,129,130]. A recent study demonstrated that MCU is involved in PD. Inhibition of MCU could rescue PINK1 deficiency-induced dopaminergic neurons loss [131]. These studies shine light on calcium channels as a target for PD therapy. A clinical trial carried out in Denmark showed that L-type calcium channel blockers are able to reduce the risk of PD (Table 1) [132]. This research suggests that targeting the MCU as a therapy for PD is hopeful.

Stroke is the third prevalent and acute neurodegenerative disorder. Glutamate-induced excitotoxicity and mitochondrial calcium overload have been proven to be the main pathogenic factors in stroke patients and stroke model animals [133,134,135]. High levels of glutamate and calcium in plasma generally indicate a poor prognosis for acute ischemic stroke patients [80,136]. Strategies targeting glutamate and calcium channels have been carried out for decades. Glutamate is an important transmitter that plays an essential role in multiple biological processes, and inhibition of glutamate release and blocking glutamate receptors returns a significant improvement in stroke, although the undesired side effects induced by blockade of the glutamate pathway hold back its therapeutic process [137,138,139]. As calcium overload is the consequence of excitotoxicity and the high serum calcium level in stroke, targeting calcium channels as a therapy seems to be a strong possibility. Calcium antagonists and calcium channel antagonists have been proven to reduce the injury induced by acute ischemic stroke in animal models and patients [140,141,142]. However, a meta-analysis recently indicated that none of the calcium and calcium channel antagonists currently developed display significant performance in acute ischemic stroke patients so far [143]. Severe side effects of calcium antagonists may be the reason for their poor effectiveness in clinical treatment, as calcium is a critical secondary messenger in mammalian cells. The discovery of the MCU provides a new target for treatment, as it is a mitochondria-specific calcium uniporter and, most importantly, mice with an outbred background can live without Mcu. According to recent studies in the rat, the MCU antagonist RU360 displays protective effects in an acute cardiac ischemic model [144]. Whether MCU antagonists can prevent stroke-induced injury is worthy of further study.

## 8. Conclusions and Future Perspectives

In the brain systems, especially in neurons, calcium overload-induced metabolic derangements and eventual cell death are not only the end of these cells but also the beginning of neuronal injury and neurodegeneration in the brain. Accumulation of excessive calcium in mitochondria causes mitochondrial dysfunction, which is found in most neurodegenerative diseases. Therefore, drugs targeting calcium or calcium channel have strong potentiality in neurodegenerative diseases therapy. A search of ClinicalTrials.gov (see: https://clinicaltrials.gov) with the keywords “calcium antagonist” and “neurodegenerative” revealed several ongoing clinical trials for AD, PD and ALS (Table 1). The MCU, the calcium uniporter on the inner membrane of mitochondria, by controlling the mitochondrial calcium uptake, is implicated in excitotoxicity, iron overload, inflammation and oxidative stress-induced mitochondrial dysfunction and cell death. The involvement of the MCU in those stress-induced cell deaths indicates that the MCU may play an essential role in neurodegenerative disorders as well, suggesting that the MCU could be a new target for disease therapy. For example, a series of studies on glutamate-induced excitotoxicity have commenced in clinical trials; however, a diverse set of drugs targeting glutamate release or glutamate receptors have so far failed due to strong side effects. Inhibition of the MCU in vitro displays significant attenuation of NMDA receptor-mediated excitotoxicity, while loss of the MCU in mice can be tolerated, suggesting that the MCU antagonist is a great potential therapy [58]. On account of this, further in vivo studies are needed to better understand the role of the MCU in neurodegeneration and the application of the MCU antagonist in neurodegenerative disorder therapy.

Targeting mitochondrial calcium has been proposed for the clinical treatment of some neurodegenerative diseases. The MCU is a better target, although further studies are still required. For example, future studies must find an effective MCU antagonist and investigate the effects of the antagonist in disease models. We believe that there will be a detailed description of the role of the MCU and the molecular signaling of MCU proteins in the pathophysiology of central nervous system diseases with more extensive studies in the future. Moreover, it would not be surprising to discover a new therapeutic strategy for neurological diseases through targeting the MCU complex or its subunits.

## Figures and Tables

**Table 1:** Calcium/calcium channel antagonist is now used in clinical trials for neurodegenerative diseases therapy.

Neurodegenerative Disorders	Drug	Target	Clinical Trial ID	Status
AD	Losartan/amlodipine	Angiotensin/calcium	NCT02913664	Phase II
	SAM-531	Calcium	NCT00745576	Phase I
	Nilvadipine/Placebo	Calcium	NCT02017340	Phase III
PD	Isradipine	Calcium channel	NCT00909545	Phase II
	Isradipine/Placebo	Calcium channel	NCT02168842	Phase III
ALS	Fasudil	Calcium	NCT01935518	Phase II

AD: Alzheimer’s disease; PD: Parkinson’s disease; ALS: amyotrophic lateral sclerosis.
